# The effects of recruitment of renal functional reserve on renal cortical and medullary oxygenation in non‐anesthetized sheep

**DOI:** 10.1111/apha.13919

**Published:** 2023-01-12

**Authors:** Alemayehu H. Jufar, Roger G. Evans, Clive N. May, Sally G. Hood, Ashenafi H. Betrie, Anton Trask‐Marino, Rinaldo Bellomo, Yugeesh R. Lankadeva

**Affiliations:** ^1^ Pre‐Clinical Critical Care Unit Florey Institute of Neuroscience and Mental Health, University of Melbourne Melbourne Victoria Australia; ^2^ Cardiovascular Disease Program, Department of Physiology Biomedicine Discovery Institute, Monash University Melbourne Victoria Australia; ^3^ Department of Critical Care Melbourne Medical School, University of Melbourne Melbourne Victoria Australia; ^4^ Melbourne Dementia Research Centre Florey Institute of Neuroscience and Mental Health, The University of Melbourne Melbourne Victoria Australia

**Keywords:** amino acid infusion, glomerular filtration rate, renal functional reserve, renal oxygenation, renal perfusion

## Abstract

**Aim:**

Recruitment of renal functional reserve (RFR) with amino acid loading increases renal blood flow and glomerular filtration rate. However, its effects on renal cortical and medullary oxygenation have not been determined. Accordingly, we tested the effects of recruitment of RFR on renal cortical and medullary oxygenation in non‐anesthetized sheep.

**Methods:**

Under general anesthesia, we instrumented 10 sheep to enable subsequent continuous measurements of systemic and renal hemodynamics, renal oxygen delivery and consumption, and cortical and medullary tissue oxygen tension (PO_2_). We then measured the effects of recruitment of RFR with an intravenous infusion of 500 ml of a clinically used amino acid solution (10% Synthamin® 17) in the non‐anesthetized state.

**Results:**

Compared with baseline, Synthamin® 17 infusion significantly increased renal oxygen delivery mean ± SD maximum increase: (from 0.79 ± 0.17 to 1.06 ± 0.16 ml/kg/min, *p* < 0.001), renal oxygen consumption (from 0.08 ± 0.01 to 0.15 ± 0.02 ml/kg/min, *p* < 0.001), and glomerular filtration rate (+45.2 ± 2.7%, *p* < 0.001). Renal cortical tissue PO_2_ increased by a maximum of 26.4 ± 1.1% (*p* = 0.001) and medullary tissue PO_2_ increased by a maximum of 23.9 ± 2.8% (*p* = 0. 001).

**Conclusions:**

In non‐anesthetized healthy sheep, recruitment of RFR improved renal cortical and medullary oxygenation. These observations might have implications for the use of recruitment of RFR for diagnostic and therapeutic purposes.

## 1 INTRODUCTION

Single nephron glomerular filtration rate (GFR) can increase in response to amino acid loading,^1^, ^2^, ^3^, ^4^, ^5^ unilateral nephrectomy,^6^, ^7^, ^8^, ^9^ or loss of functional nephrons due to acute kidney injury (AKI) or chronic kidney disease (CKD).^5^ This capacity is referred to as renal functional reserve (RFR).^5^ In a diseased kidney, recruitment of RFR maintains whole‐kidney GFR, until ~50% of functional nephrons are lost.^5^

The above concepts imply that clinically diagnosed AKI and CKD should be preceded by some decrement in RFR. This notion makes recruitment of RFR by amino acid loading a possible test for the diagnosis of subclinical kidney dysfunction.^10^, ^11^, ^12^ It also implies that recruitment of RFR has the potential to mitigate the risk of loss of renal function during clinical procedures that are injurious to the kidney like major surgery or radiocontrast administration.^13^, ^14^, ^15^ However, recruitment of RFR does not just increase GFR. It also increases the filtered sodium load and thereby renal oxygen consumption (RVO_2_), as ~80% of such RVO_2_ is due to active reabsorption of sodium.^16^ In turn, this increase in RVO_2_ may increase the risk of renal tissue hypoxia.

Renal tissue hypoxia, particularly in the renal medulla, appears central to the development of AKI arising from multiple etiologies including sepsis, cardiopulmonary bypass and radiocontrast‐induced nephropathy.^17^, ^18^, ^19^ Thus, if recruiting RFR (e.g., by amino acid loading) induces renal medullary hypoxia, this would represent a major contraindication to its use as a diagnostic or therapeutic tool. On the other hand, if RFR recruitment improved medullary oxygenation, its diagnostic and therapeutic value would be enhanced. However, the effects of recruitment of RFR on renal cortical and medullary tissue perfusion and oxygen tension (PO_2_) remain unknown.

Accordingly, we studied a clinically relevant ovine model in which renal and intrarenal perfusion and oxygenation, along with systemic hemodynamics, can be simultaneously assessed in the absence of the confounding effects of anesthesia.^20^, ^21^ In this model, we tested the hypothesis that recruitment of RFR by intravenous infusion of a clinically used proprietary mixture of amino acids (Synthamin® 17, Table 1) leads to renal cortical and/or medullary tissue hypoxia.

**TABLE 1:** Constituents of 10% Synthamin® 17

Constituent	(g/L)
L‐Leucine	7.30
L‐Isoleucine	6.0
L‐Lysine	5.8
L‐Valine	5.8
L‐Phenylalanine	5.6
L‐Histidine	4.8
L‐Threonine	4.2
L‐Methionine	4.0
L‐Tryptophan	1.8
L‐Alanine	20.7
L‐Arginine	11.5
L‐Glycine	10.3
L‐Proline	6.8
L‐Serine	5.0
L‐Tyrosine	0.4

## 2 RESULTS

Synthamin®17 (500 ml) was administered as an intravenous infusion over a 30 min period (1000 ml/h). Significant changes in renal hemodynamic variables (RBF and RVC), renal function, and oxygenation occurred before changes in systemic variables (mean arterial pressure and SVC) after commencing infusion of the amino acids (Figure S1). Thus, we present data for renal hemodynamic, renal function, and oxygenation first and for systemic variables last.

### 2.1 Renal macrocirculatory haemodynamics and kidney function

**FIGURE 1:**
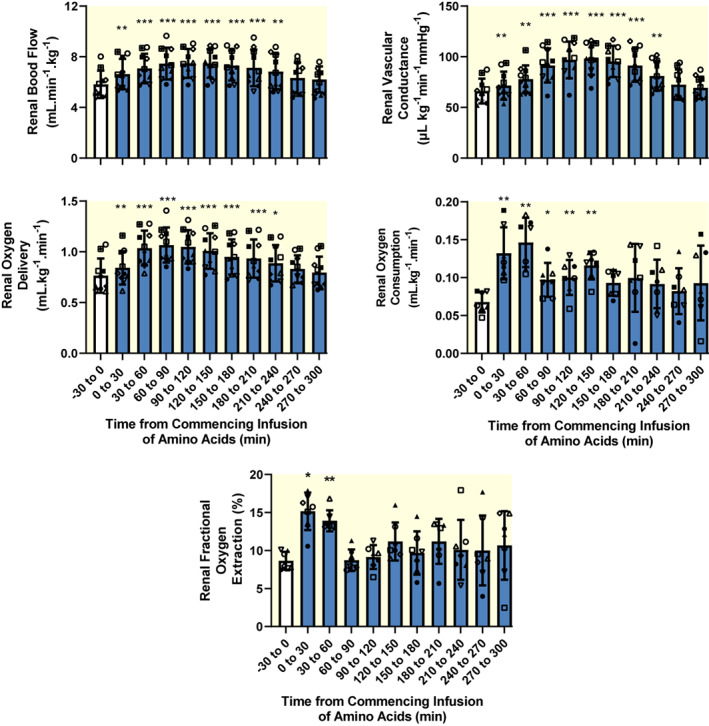
Effects of intravenous infusion of Synthamin® 17 (a proprietary mixture of amino acids) on whole‐kidney hemodynamics and oxygenation. Blue‐filled columns and error bars represent mean and standard deviation. The various symbols show data for individual sheep. There are 10 observations for all variables except for renal oxygen consumption and fractional oxygen extraction, for which *n* = 7 due to dysfunction of the renal venous cannula. Data were subjected to one‐way repeated measures analysis of variance with a Greenhouse–Geisser correction applied to the main effect of “time.” *p ≤ 0.05, **p < 0.01, ***p < 0.001 (Dunnett's test) for comparison with the baseline (−30 to 0 min). The amino acids were infused over the 0–30 min period.

Compared with the 30 min baseline period, there were significant increases in renal vascular conductance (RVC) (mean ± SD maximum increase: +34.7 ± 5.7%, 120–150 min after commencing the infusion); renal blood flow (RBF) (+29.3 ± 10.3%, 90–120 min after commencing the infusion), and renal oxygen delivery (RDO_2_; from 0.79 ± 0.17 to 1.06 ± 0.16 ml/kg/min, 60–90 min after commencing the infusion) (Figure 1). RVO_2_ also significantly increased (from 0.08 ± 0.01 to 0.15 ± 0.02 ml/kg/min, 30–60 min after commencing the infusion), as did renal fractional oxygen extraction (from 9.0 ± 1.2% to a maximum of 15.2 ± 2.5%, 0–30 min after commencing the infusion).

**FIGURE 2:**
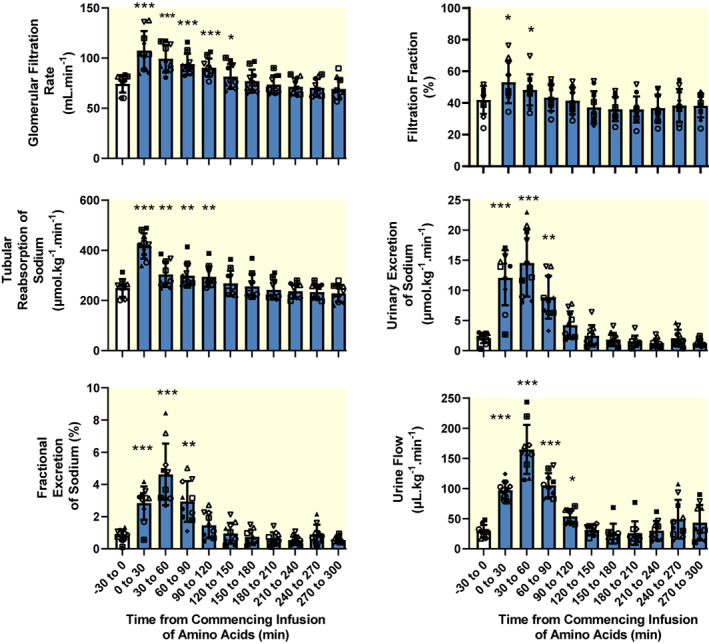
Effects of intravenous infusion of Synthamin® 17 (a proprietary mixture of amino acids) on glomerular filtration rate, filtration fraction, renal tubular reabsorption, urinary excretion, fractional excretion of sodium, and urine flow. Blue‐filled columns and error bars represent mean and standard deviation. The various symbols show data for individual sheep. There are 10 observations for all variables. Glomerular filtration rate was estimated from creatinine clearance. Filtration fraction was determined after renal plasma flow was calculated by multiplying renal blood flow by (1‐hematocrit). Hematocrit was approximated by multiplying hemoglobin by 3 (an approximate conversion factor). Data were subjected to one‐way repeated measures analysis of variance with a Greenhouse–Geisser correction applied to the main effect of “time.” **p < 0.01, ***p < 0.001 (Dunnett's test) for comparison with the baseline (−30 to 0 min). The amino acids were infused over the 0–30 min period.

Glomerular filtration rate, as estimated from creatinine clearance, increased significantly from its baseline level (maximum increase: +45.2 ± 18.5%, 0–30 min after commencing the infusion; Figure 2). Filtration fraction also significantly increased from a baseline of 42.0 ± 8.8% to a maximum of 53.2 ± 13.3%, 0–30 min after commencing the infusion. Tubular reabsorption of sodium (+40.3 ± 3.6%, 0–30 min after commencing the infusion) and urinary excretion of sodium (+84.0 ± 10.2%, 30–60 min after commencing the infusion) significantly increased due to both the increase in GFR and to a marked increase in fractional excretion of sodium (from a baseline of 0.84 ± 0.35% to a maximum of 4.61 ± 1.91%, 30–60 min after commencing the infusion). Urine flow also increased significantly (maximum increase: +81.2 ± 8.4%, 30–60 min after commencing the infusion).

### 2.2 Cortical and medullary microcirculatory perfusion and oxygenation

**FIGURE 3:**
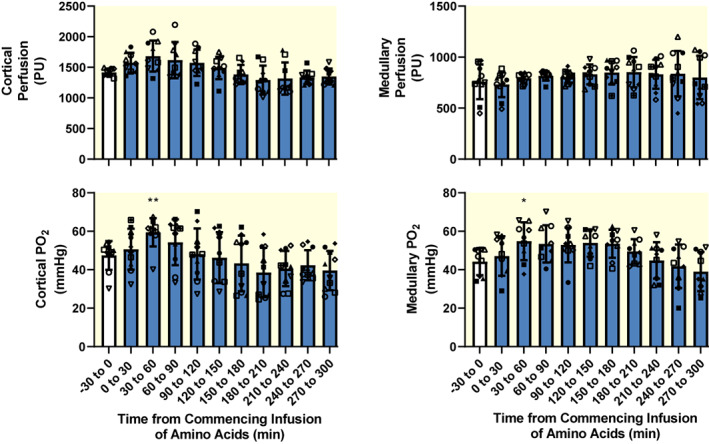
Effects of intravenous infusion of Synthamin® 17 (a proprietary mixture of amino acids) on renal tissue perfusion and oxygen tension. Blue‐filled columns and error bars represent mean and standard deviation. The various symbols show data for individual sheep. PO_2_, partial pressure of oxygen; PU, perfusion unit. *n* = 10 for cortical PO_2_ and medullary perfusion. Due to dysfunction of fiber‐optic probes, *n* = 9 for cortical perfusion and medullary PO_2_. Data were subjected to one‐way repeated measures analysis of variance with a Greenhouse–Geisser correction applied to the main effect of “time.” *p ≤ 0.05, **p < 0.01 (Dunnett's test), for comparison with the baseline (−30 to 0 min). The amino acids were infused over the 0–30 min period.

Amino acids infusion did not significantly change renal cortical or medullary tissue Doppler flux, an index of microvascular perfusion (Figure 3). However, renal cortical tissue oxygen tension (PO_2_) increased significantly (maximum of +26.4 ± 1.1%, 30–60 min after commencing the infusion), as did medullary tissue PO_2_ (+26.9 ± 2.8%, 30–60 min after commencing the infusion).

### 2.3 Systemic haemodynamics and oxygenation

**FIGURE 4:**
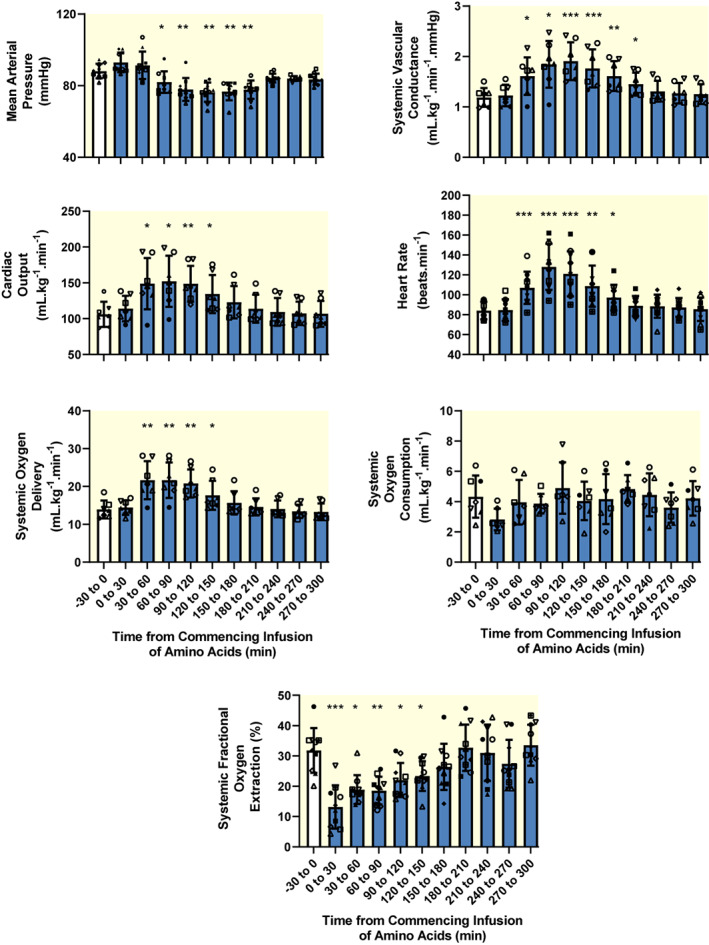
Effects of intravenous infusion of Synthamin 17® (a proprietary mixture of amino acids) on heart rate, cardiac output and systemic hemodynamics and oxygenation. Blue‐filled columns and error bars represent mean and standard deviation. The various symbols show data for individual sheep. *n* = 10 for heart rate, mean arterial pressure, and systemic fractional oxygen extraction. *n* = 7 for cardiac output, systemic vascular conductance, and systemic oxygen delivery and systemic oxygen consumption. Systemic vascular conductance was determined by dividing cardiac output by the product of body weight and mean arterial pressure. Systemic oxygen delivery was calculated by multiplying cardiac output by the oxygen content in the arterial blood. Data were subjected to one‐way repeated measures analysis of variance with a Greenhouse–Geisser correction applied to the main effect of “time.” *p ≤ 0.05, **p < 0.01, ***p < 0.001 (Dunnett's test) for comparison with the baseline (−30 to 0 min). The amino acids were infused over the 0–30 min period.

Compared with the 30 min baseline period prior to commencing the amino acid infusion, heart rate increased (from a baseline of 83.0 ± 8.0 beats/min to a maximum of 128.0 ± 23.0 beats/min, 60–90 min after commencing the infusion), cardiac output (CO) increased (maximum increase: +42.8 ± 20.0%, 60–90 min after commencing the infusion) and mean arterial pressure fell (from a baseline of 88.0 ± 4.0 mm Hg to a nadir of 76.4 ± 5.4 mm Hg, 120–150 min after commencing the infusion) (Figure 4). Thus, systemic vascular conductance increased (SVC; maximum increase: +48.3 ± 10.8%, 90–120 min after commencing the infusion). The increase in CO was accompanied by a marked increase in systemic oxygen delivery (SDO_2_) (maximum increase: +48.7 ± 16.3%, 60–90 min after commencing the infusion). However, systemic VO_2_ was not significantly altered, and systemic fractional oxygen extraction was markedly reduced (from a baseline level of 30.2 ± 5.6% to a nadir of 16.0 ± 7.2%, 30–60 min after commencing the infusion).

Infusion of Synthamin® was followed by a small degree of hemoconcentration, with arterial hemoglobin concentration increasing by a maximum of +8.5 ± 5.9% (30–60 min after commencing the infusion; Table 2). Consequently, arterial oxygen content increased by a maximum of +8.9 ± 6.5% (30–60 min after commencing the infusion). Infusion of Synthamin® was followed by sustained increases in mixed venous PO_2_ and oxygen saturation of hemoglobin (Table S1). Additional arterial blood biochemical changes are presented in Table 2.

**TABLE 2:** Arterial blood oximetry and chemistry

Variable	Time (in min) from Commencement of IV Infusion of Synthamin® 17										
	1. Baseline	2. 0–30	3. 30–60	4. 60–90	5. 90–120	6. 120–150	7. 150 –180	8. 180–210	9. 210–240	10. 240–270	11. 270–300
PO_2_ (mm Hg)	100.7 ± 5.7	106.5 ± 5.5	111.6 ± 8.1***	107.7 ± 7.4	108.1 ± 9.2*	106.9 ± 9.8	106.5 ± 7.4	104.6 ± 6.9	104.3 ± 12.2	101.1 ± 8.3	106.6 ± 8.5
SO_2_ (%)	96.0 ± 1.2	96.6 ± 0.9	96.8 ± 1.0	96.4 ± 1.2	96.4 ± 1.1	96.2 ± 1.4	96.4 ± 1.4	96.1 ± 1.1	96.0 ± 1.1	95.7 ± 1.3	96.4 ± 1.1
Hb (g/dl)	9.7 ± 0.6	9.6 ± 0.7	10.5 ± 0.5**	10.4 ± 0.4**	10.3 ± 0.4*	10.0 ± 0.5	9.48 ± 0.5	9.6 ± 0.5	9.5 ± 0.4	9.4 ± 0.6	9.3 ± 0.5*
Oxygen content (ml O_2_/dl)	13.2 ± 0.9	13.3 ± 0.9	14.5 ± 0.8**	14.3 ± 0.6**	14.6 ± 0.6*	13.7 ± 0.6	13.0 ± 0.6	13.2 ± 0.6	13.0 ± 0.7	12.8 ± 0.8	12.7 ± 0.7*
PCO_2_ (mm Hg)	32.0 ± 2.2	31.5 ± 2.3	31.7 ± 3.0	32.4 ± 2.0	32.8 ± 2.2	33.4 ± 2.8	33.0 ± 3.7	33.6 ± 3.7	32.6 ± 2.7	33.3 ± 2.7	32.0 ± 2.5
pH	7.53 ± 0.02	7.52 ± 0.01	7.53 ± 0.02	7.53 ± 0.02	7.53 ± 0.02	7.52 ± 0.02	7.53 ± 0.03	7.51 ± 0.03	7.52 ± 0.03	7.50 ± 0.03**	7.52 ± 0.03
Lactate (mmol/L)	0.65 ± 0.25	1.10 ± 0.15***	1.16 ± 0.24***	1.16 ± 0.30***	1.11 ± 0.28***	1.09 ± 0.29**	1.01 ± 0.32*	0.91 ± 0.35	0.82 ± 0.26	0.71 ± 0.23	0.65 ± 0.16
Sodium (mmol/L)	143.8 ± 4.6	130.0 ± 4.4***	134.8 ± 6.5*	135.9 ± 3.2**	137.4 ± 4.3*	138.2 ± 3.5	138.6 ± 5.4	136.6 ± 3.3**	136.4 ± 2.9**	137.5 ± 4.0**	138.8 ± 2.3**
Potassium (mmol/L)	4.08 ± 0.24	3.59 ± 0.28**	3.37 ± 0.28***	3.14 ± 0.27***	3.11 ± 0.25***	3.16 ± 0.25***	3.34 ± 0.41**	3.32 ± 0.29***	3.45 ± 0.36**	3.49 ± 0.32**	3.53 ± 0.37**
Chloride (mmol/L)	109.7 ± 3.5	100.3 ± 2.5***	104.4 ± 4.2*	105.1 ± 4.0**	107.2 ± 4.4*	107.9 ± 3.5	109.2 ± 3.5	106.3 ± 2.6*	106.2 ± 2.8*	107.4 ± 3.1	107.1 ± 2.4
Calcium (mmol/L)	1.12 ± 0.11	1.06 ± 0.05	1.07 ± 0.12	1.13 ± 0.06	1.11 ± 0.10	1.15 ± 0.11	1.13 ± 0.12	1.16 ± 0.10	1.14 ± 0.05	1.15 ± 0.07	1.15 ± 0.08
Bicarbonate (mmol/L)	26.5 ± 2.5	25.7 ± 2.2*	26.5 ± 2.5	26.9 ± 2.2	27.3 ± 2.8**	27.2 ± 2.8	27.2 ± 2.6	26.7 ± 2.5	26.2 ± 2.8	25.9 ± 2.7	25.8 ± 2.8
Plasma Cr (μmol/L)	88.6 ± 12.5	90.2 ± 12.9	86.0 ± 11.3	82.6 ± 12.4**	80.0 ± 12.1**	77.4 ± 11.8**	77.2 ± 11.6**	76.4 ± 11.2**	76.8 ± 11.9**	76.7 ± 11.5**	76.2 ± 10.4***

*Note*: Data are expressed as mean and standard deviation. Blood oxygen content was calculated as (0.0139 X [Hb] X SO_2_) + (0.003 X PO_2_). *n* = 10 for all variables. Data were subjected to one‐way repeated measures analysis of variance with a Greenhouse–Geisser correction applied to the main effect of “time.” Within‐animal pairwise comparisons were performed using Dunnett's test. The baseline measurements were conducted for a period of 30 min. Synthamin® 17 (a proprietary mixture of amino acids) was then infused over the 0–30 min period.

Abbreviations: Cr, creatinine; PO_2_, partial pressure of oxygen; PCO_2_, partial pressure of carbon dioxide; SO_2_, saturation of hemoglobin with oxygen; Hb, blood hemoglobin concentration.

* *p* ≤ 0.05

** *p* < 0.01

*** *p* < 0.001 (Dunnett's test) for comparison with the baseline.

## 3 DISCUSSION

In a clinically relevant non‐anesthetized large mammalian model, as expected, we observed markedly increased RBF, RDO_2_, GFR, RVO_2_, and renal fractional oxygen extraction in response to recruitment of RFR with intravenous infusion of a clinically approved proprietary mixture of amino acids (Synthamin® 17). In this setting, however, for the first time to our knowledge, we demonstrated that recruitment of RFR also increased renal cortical and medullary tissue PO_2_.

In our current study, the percentage increase in GFR (~45%) in response to recruitment of RFR was similar to, or greater than that reported from previous studies.^1^, ^2^, ^3^, ^22^, ^23^, ^24^ Our current finding, of increased renal medullary tissue PO_2_ during amino acid loading in conscious sheep, contrasts with the only previous relevant study in this field, which demonstrated reduced renal medullary tissue PO_2_ after infusion of a single amino acid, glycine, in anesthetized rats.^25^ This discrepancy in medullary tissue PO_2_ may be, at least in part, due to the impact of infusing a single amino acid in rats compared with administering a clinically used mixture of 15 amino acids for recruitment of RFR in sheep. Moreover, it could also be due to the difference in the methods used to determine renal tissue PO_2_, the impact of anesthesia and species variations. Anesthesia has been found to be associated with reduced RBF (and thus RDO_2_) in rats,^26^ rabbits,^27^ sheep,^20^, ^21^, ^28^ and humans.^29^ Anesthesia might also be expected to alter renal medullary oxygen consumption (and thus increase the risk of renal medullary hypoxia) through an impact on tubular sodium reabsorption; activation of renal sympathetic nerve activity and the renin‐angiotensin system.^21^, ^30^, ^31^, ^32^ Importantly, our findings are clinically relevant to awake non‐anesthetized patients, who are those typically given amino acids to recruit RFR, either as a test for subclinical renal dysfunction^10^, ^11^, ^12^ or as a therapy to promote increased GFR in states of renal dysfunction.^33^ Currently, a larger multicenter, randomized clinical trial is underway (Clinical Trials. gov identifier: NCT03709264) to determine whether infusion of amino acids during and after cardiac surgery requiring cardiopulmonary bypass reduces the incidence of post‐operative AKI.

The time‐course of changes in renal function and oxygenation differed from those of systemic and renal hemodynamics. GFR, filtration fraction, sodium reabsorption, RVO_2_ and renal fractional oxygen extraction all increased to their maximum within 60 min of commencing the amino acid infusion. In contrast, the increases in RBF, RDO_2_, and RVC, and the increases in CO, DO_2_, and SVC, together with the fall in mean arterial pressure, occurred over a longer timescale, reaching their maximum between 60 and 150 min after the infusion commenced. The consequence of these differences in the time‐course of changes in the determinants of RVO_2_ (early) and RDO_2_ (later) might promote renal tissue hypoxia in the early phase and hyperoxia in the later phase. However, renal cortical and medullary tissue PO_2_ increased within the first 60 min after commencing the infusion of Synthamin®, when RVO_2_ was increased rather than decreased. Furthermore, the maximal increases in RBF and RDO_2_ were not associated with further increases in renal tissue PO_2_. Thus, our findings suggest that intrarenal tissue oxygenation after infusion of Synthamin® 17 is poorly predicted from global RBF, RDO_2_, RVO_2_.

Our previous findings in sheep also support the notion that renal medullary and/or cortical tissue oxygenation can be maintained despite mismatched changes in RDO_2_ and RVO_2_.^34^, ^35^, ^36^, ^37^, ^38^, ^39^, ^40^, ^41^ The mechanisms underlying this apparent dissociation between global and regional kidney oxygenation are not completely understood. They might include diffusive shunting of oxygen and/or carbon dioxide, in both the cortex and medulla,^40^ and the impact of differences in the efficiency of utilization of oxygen for sodium reabsorption along the various nephrons segments.^34^

The baseline filtration fraction measured in the current study (~42%) was greater than that previously reported in sheep (~20%).^42^ This discrepancy could be due to the difference in methods used to determine renal plasma flow. In our current study, renal plasma flow was calculated by multiplying RBF by (1‐hematocrit) after hematocrit was approximated by multiplying the blood concentration of hemoglobin (in mg/dl) by 3 (an approximate conversion factor). However, in the previous study hematocrit was measured directly and renal plasma flow was determined via clearance of para‐aminohippurate. Thus, absolute values of filtration fraction presented herein may be overestimates. Nevertheless, we are confident that our data faithfully reflect the pattern of changes in filtration fraction in response to amino acid infusion.

Amino acid loading increases the filtered load of amino acids and co‐reabsorption of NaCl with amino acids in the proximal tubule. This reduces delivery of NaCl to the macula densa.^5^ In response to this, the macula densa releases paracrine factors such as nitric oxide and prostaglandins, which in turn reduce the responsiveness of tubulo‐glomerular feedback. This process leads to dilation of the afferent arteriole^5^ and consequent increases in RBF and GFR. Amino acid loading also appears to increase the release of glucagon from α‐cells of the pancreas.^24^, ^43^ Glucagon plays a permissive role on the effects of nitric oxide and prostaglandins on tubulo‐glomerular feedback.^5^ Reduced plasma oncotic pressure can increase GFR when extracellular fluid volume is expanded.^44^ However, this mechanism is unlikely to have contributed to the increased GFR in our current study, since the amino acid infusion was followed by a small degree of hemoconcentration rather than hemodilution. Given the observed increase in filtration fraction, it is also possible that the amino acid infusion led to release of local factor (s), which preferentially constrict the efferent arterioles, thus leading to increased glomerular capillary hydrostatic pressure. This proposition merits investigation.

Despite significantly increased RBF following infusion of amino acids, using laser Doppler flowmetry we did not detect significant changes in renal cortical or medullary tissue perfusion. This could be attributable to technical differences between transit‐time ultrasound flowmetry and laser Doppler flowmetry, the phenomenon of vascular shunting, or both. Transit‐time flowmetry measures absolute RBF with a high level of accuracy.^45^ In contrast, laser Doppler flowmetry provides an index of blood flow in the microcirculation with an actual sampling volume of only ~1 mm^3^ of tissue. Furthermore, in tissues where perfusion is high (e.g., in the kidney), laser Doppler flowmetry measures mean erythrocyte velocity rather than an absolute value of blood flow^46^ and is, therefore, insensitive to changes in the number of perfused capillaries. There is evidence for convective shunts in the renal circulation.^47^, ^48^ However, whether such shunts are further enhanced by amino acid loading has not been determined.

As in previous studies of the effects of amino acid loading, we observed increased diuresis^14^, ^49^ and natriuresis.^50^, ^51^, ^52^ Under physiological conditions, filtered amino acids are nearly completely reabsorbed in the proximal tubule by sodium‐dependent cotransport mechanisms.^53^ However, when the filtered load is more than the reabsorption capacity of the proximal tubule, osmotic diuresis occurs, leading to increased urine flow and loss of electrolytes. This likely partly explains the reductions in plasma concentrations of sodium, potassium and chloride after infusion of Synthamin®.

Our observation that natriuresis occurred after infusion of Synthamin®, despite no significant change in, or a significantly reduced, MAP is not consistent with the notion that renal sodium excretion is principally controlled by arterial pressure (pressure natriuresis). The concept that pressure natriuresis is vital for the regulation of arterial pressure has recently been questioned.^54^ The basis of the concept is that, when arterial pressure and thus renal perfusion pressure increases, renal excretion of sodium increases leading to a decrease in blood volume and thus restoration of blood pressure back to normal.^55^ The opposite occurs when arterial pressure decreases. Theoretically, therefore, pressure natriuresis can reverse any change in arterial pressure regardless of the cause or magnitude. The corollary to this concept is that impairment of the pressure natriuresis response leads to sustained changes in arterial blood pressure including hypertension. However, the role of pressure natriuresis in control of arterial pressure has been challenged by accumulating evidence that shows marked changes in the renal excretion of sodium despite no significant change in arterial pressure.^54^ For example, in dogs, infusion of either isotonic or hypertonic saline at rates of 6 and 20 μmol/min/kg markedly increased renal excretion of sodium despite no significant change in arterial pressure.^56^ Similarly, in humans, either administration of a high sodium diet^57^ or infusion of isotonic^58^ or hypertonic saline^58^ was associated with markedly increased renal excretion of sodium despite no significant change (after infusion of isotonic saline and high sodium diet) or significantly reduced (after infusion of hypertonic saline) arterial pressure. It has, therefore, been suggested that mechanisms (other than pressure natriuresis) such as neurohumoral factors (e.g., the renin‐angiotensin system, renal sympathetic nerve activity) are the primary controllers of renal sodium excretion.^54^, ^55^, ^59^ Our current physiological experimental design did not allow us to investigate the mechanisms mediating natriuresis in response to amino acid infusion, which merits further study.

The increased SVC after infusion of Synthamin® is attributable to the combined effects of increased RVC and dilation of other systemic resistance vascular beds (as evidenced by a greater increase in SVC (~43.0%) than RVC (~31.0%)). Because Synthamin® contains the amino acid, L‐ arginine, which is a substrate for vascular nitric oxide formation, it can lead to dilation of resistance vascular beds and thus to an increase in SVC.^60^ Indeed, in previous studies in rodents, it has been demonstrated that amino acid‐induced renal vasodilation and glomerular hyperfiltration were at least blunted by administration of inhibitors of nitric oxide synthase.^61^, ^62^ Nevertheless, future studies are warranted to assess the activity of neurohormonal factors that might mediate or modulate the systemic and renal vasodilatory effects of Synthamin® infusion.

Decreases in arterial pressure induce baroreceptor‐mediated activation of sympathetic outflow to the heart and peripheral vasculature and inhibition of parasympathetic outflow to the heart.^63^ The resultant effects of these changes on autonomic function are increased heart rate and stroke volume, and constriction of systemic blood vessels (and thus a decrease in SVC), which together leads to an increase in and thus restoration of arterial pressure back to normal. However, in the current study, SVC increased, and MAP decreased despite increases in heart rate and CO after infusion of Synthamin®, indicating that direct vasodilator effects overrode the effects baroreceptor‐mediated increases in heart rate, CO, and vasoconstriction.

Currently available diagnostic tests for AKI and CKD can neither detect subclinical kidney dysfunction nor be used as appropriate markers for prognostication of the progression of subclinical to clinical stages. Moreover, available treatments for AKI and CKD are costly, of questionable efficacy, and not without risk. Thus, our findings suggest that it may be possible to use RFR as a diagnostic tool in healthy subjects, which may have clinical implications for its utility to stratify patients according to their risk of AKI before major surgery or other medical procedures associated with iatrogenic AKI. However, future studies are warranted to test the safety and efficacy of recruitment of RFR as a diagnostic tool in clinically relevant animal models of clinical and/or subclinical CKD and both during and after recovery from various forms of AKI, prior to clinical translation.

The long‐term effects of recruitment of RFR on the kidney have not been adequately investigated. In humans, the increase in single‐kidney GFR after unilateral nephrectomy (considered to represent RFR) is maintained as measured later at various times after kidney donation. A greater increase in estimated GFR from its pre‐donation value 3 months after unilateral nephrectomy (≥16 ml/min 1.73 m^−2^) was found to be associated with better kidney function 10 years after the kidney donation (the increased GFR was maintained and was not associated with proteinuria).^64^ Similarly, estimated GFR was found to be increased by ~33% from pre‐donation values 3 months after unilateral nephrectomy and was well maintained 2 years after in kidney donors.^8^ In another study, estimated GFR in donors 1 year after unilateral nephrectomy was found to be 24%–75% greater than its pre‐donation value.^9^ Thus, at least in the context of the chronic effects of kidney donation, recruitment of RFR appears free of detectable detrimental effects on long‐term renal function.

Strengths of our study include the use of a clinically relevant large animal model and the absence of the potentially confounding effects of general anesthesia. The percentage increase in GFR in response to recruitment of RFR in our study is similar to or greater than that reported from previously studies.^1^, ^2^, ^3^, ^22^, ^23^, ^24^ Limitations include the fact that we studied young and healthy animals. Thus, our findings are more relevant to young healthy individuals than older healthy individuals and patients who have AKI or CKD or are suspected of having subclinical kidney dysfunction. The other limitation of our study is the lack of a time‐control group. However, we have previously reported that systemic hemodynamics and global and regional kidney perfusion and oxygenation are stable over a 24‐h period in healthy non‐anesthetized sheep.^36^, ^65^ We also acknowledge the limitations of laser Doppler flowmetry, which measures erythrocyte velocity rather than the actual blood flow in highly perfused organs such as the kidneys.^46^ We also do not have information regarding changes in components of the renin‐angiotensin system during Synthamin® infusion. However, previous studies in dogs^66^ and humans^67^ have not demonstrated changes in plasma renin activity in response to intravenous or oral amino acid loading. Lastly, due the pharmacokinetic differences between oral and intravenous administration of amino acids, the magnitude and underlying mechanisms of the RFR observed in the current study may differ from those recruited by oral administration of amino acids and/or proteins.

In conclusion, in a clinically relevant non‐anesthetized large mammalian model, recruitment of RFR with intravenous infusion of a clinically approved proprietary mixture of amino acids (Synthamin® 17) increased cortical and medullary tissue PO_2_. Our findings might have implications for use of the RFR challenge as a “physiological biomarker” for the detection of subclinical kidney dysfunction and/or as a prophylactic or therapeutic tool in various forms of AKI.

## 4 MATERIALS AND METHODS

### 4.1 Ethics

These experimental studies were conducted after approval by the Animal Ethics Committee of the Florey Institute of Neuroscience and Mental Health under the guidelines of the National Health and Medical Research Council of Australia. All studies were conducted according to the Animal Research: Reporting of In Vivo Experiments (ARRIVE) criteria.^68^ Ten female non‐pregnant Merino ewes (1.5–2.0 years of age), with a mean body weight of 44.70 ± 0.03 kg mean ± SD, were used. The sheep were housed in individual metabolic cages, with each sheep allowed ad libitum access to water and 800 g of oaten chaff food per day.

### 4.2 Surgical preparation

Two separate preparative surgical procedures, for instrumentation of the sheep, were done under general anesthesia. In both, anesthesia was induced with sodium thiopentone (15 mg/kg; Jurox) and maintained with isoflurane (2.0%–2.5%; Isoflo, Zoetis). In the first procedure, as previously described,^69^ the pericardium was opened via a left thoracotomy and a transit‐time flow probe (20 mm; Transonic Systems) was placed around the pulmonary artery for measurement of CO.^69^, ^70^

The second surgical procedure was performed after a 2‐week recovery period. As described in detail previously,^65^, ^70^, ^71^ a transit‐time flow probe was placed around the left renal artery, for measurement of RBF, and custom‐built fiber‐optic probes (450 μm outer diameter; CP‐004‐001 Oxford Optronix), with 20 mm of optical fiber extending from the outer sheath, were inserted into the renal cortex and medulla for measurement of renal tissue perfusion, oxygenation, and temperature. Each probe contains a dual‐fiber laser Doppler probe for estimation of local tissue perfusion by measurement of laser Doppler flux, a single‐fiber fluorescence optode for measurement of tissue PO_2_, and a thermocouple for measurement of tissue temperature.^70^, ^72^, ^73^, ^74^ Prior to insertion of probes into the renal cortex and medulla, guiding routes were made by insertion of a 25‐gauge needle (514‐μm outer diameter). The renal cortical probe was then inserted 20 mm at an angle of 10° so its tip was 2–3 mm below the renal capsule. The renal medullary probe was inserted at 60° so its tip was 6–10 mm below the renal capsule. At post‐mortem, we confirmed that the tips of all probes were within these ranges of depths below the renal capsule.

During the second surgical procedure, the carotid artery and renal vein were cannulated for measurement of arterial pressure, heart rate and for sampling of arterial and renal venous blood. The jugular vein was cannulated for sampling of mixed venous blood and infusion of fluids. A urinary catheter (Foley size 14 French, 30 ml; Euromedical) was inserted and subsequently connected to a fraction collector for measurement of urine flow and collection of urine samples.

For both surgical procedures, each sheep received intramuscular injections of antibiotic (procaine penicillin 900 mg; Ilium, Troy Laboratories) and analgesic (flunixin meglumine 50 mg; Norbrook) both pre‐operatively (just prior to surgery) and post‐operatively (every 24 h for 2 days). Experiments were conducted after 4 days of recovery from the second surgical procedure.

### 4.3 Experimental measurements

Variables including CO, arterial pressure, RBF, tissue perfusion, and PO_2_ were recorded digitally as previously described.^65^, ^69^ Arterial, mixed venous, and renal venous blood samples for oximetry and blood chemistry (ABL Systems 625) were collected at the end of each 30‐min experimental period (see protocol below). Urine flow was measured volumetrically. The concentrations of creatinine and sodium in arterial plasma and urine were measured in a hospital pathology laboratory. In clinical practice, GFR is usually estimated from measurement of the plasma concentration of creatinine^75^ (e.g., by using the Cockcroft–Gault equation)^76^ or renal creatinine clearance.^75^ In the current study, we estimated GFR from creatinine clearance, as the product of the urinary concentration of creatinine and urine flow divided by the plasma concentration of creatinine at the end of each 30‐min experimental period.

We assessed the potential for expansion of the extracellular fluid volume, during and after amino acid infusion, to dilute plasma creatinine and thus confound measurement of creatinine clearance. Plasma creatinine concentration did not significantly change across the course of the experiment (Figure S2). Therefore, creatinine clearance was calculated using the plasma creatinine concentration at the end of each experimental period.

### 4.4 Experimental protocol

All experiments were conducted while the sheep were conscious and unrestricted in its metabolic cage. The experiment was divided into 11 sequential 30‐min experimental periods. The first 30‐min experimental period served as baseline. This was followed by a 30‐min period of intravenous infusion of a proprietary mixture of L‐amino acids (500 ml of 10% Synthamin® 17, 50 g. 500 ml^−1^, Electrolyte Free; Baxter Healthcare, Table 1) at a rate of 1000 ml/h. A further nine 30‐min experimental periods followed. We calculated the dose of Synthamin® 17 based on a previous clinical study in which 100 g of Synthamin® 17 was used to recruit RFR in humans with a mean body weight of ~90 kg.^14^ Thus, we used 50 g of Synthamin® 17 for sheep with a mean body weight of ~45 kg.

At the end of the experiment, the sheep were euthanized with sodium pentobarbitone administered intravenously (20 mg/kg; Lethobarb, Virbac).

### 4.5 Statistical analysis

All data passed the Kolmogorov–Smirnov (*n* ≥ 5) normality test. Data are expressed as mean ± SD. Data were first subjected to one‐way repeated measures analysis of variance (anova) with a Greenhouse–Geisser correction applied to the main effect of “time.”^77^ Within‐animal pairwise comparisons between each time period and the baseline period were then performed using Dunnett's test.^78^ Two‐sided *p* ≤ 0.05 was considered statistically significant. All analyses were performed using GraphPad Prism 8 (GraphPad Software).

## AUTHOR CONTRIBUTIONS

YRL, CNM, AHJ, RB, and RGE conceived and designed the research; AHJ, YRL, SH, AHB, and ATM, performed the experiments. AHJ analyzed data and drafted the manuscript; AHJ, RGE, YRL, and CNM interpreted the data. All authors edited and revised the manuscript and approved the final version.

## FUNDING INFORMATION

This study was supported by the National Health and Medical Research Council of Australia (GNT1122455, GNT1185777), the Victorian Government Operational Infrastructure Support Grant, and the National Heart Foundation of Australia (101853, 105666). AHJ was supported by a postgraduate scholarship from Monash University.

## CONFLICT OF INTEREST

All authors declare no conflicts of interest.

## Supporting information


Appendix S1.

## Data Availability

The data underlying this article will be shared on reasonable request to the corresponding author.
