# Supplementary material for: The effects of recruitment of renal functional reserve on renal cortical and medullary oxygenation in non‐anesthetized sheep
Source: Acta Physiol (Oxf). 2023 Jan 12;237(4):e13919. doi: 10.1111/apha.13919 (PMC10909474; doi:10.1111/apha.13919)
Supplement: Supplementary file 1 — Appendix S1. [file APHA-237-e13919-s001.docx]

**ONLINE SUPPLEMENT**

**The effects of recruitment of renal functional reserve on**

**renal cortical and medullary oxygenation in non-anaesthetised sheep**

Alemayehu H. Jufar^1,2^, Roger G. Evans^1,2^, Clive N. May^1,3^, Sally G. Hood^1^, Ashenafi H. Betrie^1,4^, Anton Trask-Marino^1^, Rinaldo Bellomo^1,3^ & Yugeesh R. Lankadeva^1,3^

^1^Pre-clinical Critical Care Unit, Florey Institute of Neuroscience and Mental Health, University of Melbourne, Melbourne, Victoria, Australia.

^2^Cardiovascular Disease Program, Biomedicine Discovery Institute and Department of Physiology, Monash University, Melbourne, Victoria, Australia.

^3^Department of Critical Care, Melbourne Medical School, University of Melbourne, Victoria, Australia.

^4^Melbourne Dementia Research Centre, Florey Institute of Neuroscience and Mental Health, The University of Melbourne, Victoria, Australia.

**Running title:** Renal oxygenation during amino acid loading

***Author for correspondence:**

Yugeesh R Lankadeva

Pre-clinical Critical Care Unit

Florey Insitute of Neuroscience and Mental Health

30 Royal Parade, University of Melbourne, Parkville, Victoria, 3052

Email: [yugeesh.lankadeva@florey.edu.au](mailto:yugeesh.lankadeva@florey.edu.au)

Phone: +61 (3) 8334 0417 Mobile: +61 (4) 0664 9976

| **Variable** | **1. Baseline** | **Time (in min) from Commencement of IV Infusion of Synthamin® 17** | | | | | | | | | |
| --- | --- | --- | --- | --- | --- | --- | --- | --- | --- | --- | --- |
|  |  | 1. **0 – 30** | 1. **30 – 60** | **4. 60 – 90** | 1. **90 – 120** | 1. **120 – 150** | 1. **150 – 180** | 1. **180 – 210** | 1. **210 – 240** | 1. **240 – 270** | 1. **270 – 300** |
| **Mixed Venous**  **Blood** | |  |  |  |  |  |  |  |  |  |  |
| PO_2_ (mmHg) | 45.67 ± 6.17 | 57.86 ± 6.05******* | 59.92 ± 5.56******* | 56.37 ± 7.03***** | 56.05 ± 6.39***** | 52.91 ± 3.93***** | 51.6 ± 6.2***** | 49.2 ± 12.1 | 47.5 ± 5.7 | 49.0 ± 4.2 | 44.6 ± 3.7 |
| SO_2_ (%) | 66.69 ± 8.05 | 82.7 ± 3.76******* | 82.6 ± 2.51******* | 80.53 ± 2.90****** | 76.81 ± 4.76***** | 75.10 ± 3.56****** | 72.2 ± 6.8 | 67.2 ± 8.6 | 67.7 ± 7.6 | 69.5 ± 5.9 | 65.8 ± 5.4 |
| P_50_ (mmHg) | 35.0 ± 4.5 | 32.2 ± 3.3 | 33.6 ± 2.4 | 33.2 ± 3.0 | 35.2 ± 4.2 | 35.1 ± 2.7 | 36.0 ± 4.7 | 37.3 ± 7.3 | 35.7 ± 2.5 | 34.5 ± 2.3 | 35.0 ± 3.0 |
| Hb (g/dL) | 9.61 ± 0.73 | 9.51 ± 0.68 | 10.19 ± 1.01 | 10.27 ± 0.32***** | 10.09 ± 0.44 | 9.74 ± 0.63 | 9.4 ± 0.6 | 9.3 ± 0.6 | 9.4 ± 0.7 | 9.4 ± 0.5 | 9.1 ± 0.7 |
| Oxygen Content  (mL O_2_/dL) | 9.01 ± 0.86 | 11.11 ± 0.99****** | 11.87 ± 1.10****** | 11.67 ± 0.57******* | 10.94 ± 0.86****** | 10.32 ± 0.67****** | 9.6 ± 1.2 | 8.8 ± 1.1 | 8.9 ± 0.9 | 9.2 ± 0.8 | 8.4 ± 0.6***** |
| PCO_2_ (mmHg) | 35.24 ± 3.12 | 35.15 ± 2.90 | 36.27 ± 3.82 | 35.53 ± 2.53 | 36.96 ± 3.38 | 37.37 ± 3.72***** | 37.3 ± 3.7***** | 37.3 ± 3.7 | 36.8 ± 3.5 | 35.9 ± 2.5 | 36.0 ± 3.0 |
| pH | 7.49 ± 0.03 | 7.49 ± 0.03 | 7.48 ± 0.05 | 7.49 ± 0.02 | 7.49 ± 0.01 | 7.48 ± 0.02 | 7.48 ± 0.02 | 7.47 ± 0.03 | 7.47 ± 0.03 | 7.48 ± 0.03 | 7.46 ± 0.04 |
| Lactate (mM) | 0.84 ± 0.49 | 1.18 ± 0.20 | 1.28 ± 0.28***** | 1.47 ± 0.82****** | 1.31 ± 0.33****** | 1.23 ± 0.32***** | 1.23 ± 0.29***** | 1.13 ± 0.43 | 0.99 ± 0.34 | 0.99 ± 0.54 | 0.87 ± 0.29 |
| **Renal Venous**  **Blood** | |  |  |  |  |  |  |  |  |  |  |
| PO_2_ (mmHg) | 61.05 ± 5.05 | 62.49 ± 9.48 | 66.99 ± 5.55 | 67.99 ± 5.48***** | 67.53 ± 6.88 | 65.66 ± 5.77***** | 66.3 ± 6.0 | 64.5 ± 5.4 | 65.5 ± 4.7 | 62.8 ± 4.6 | 63.1 ± 4.3 |
| SO_2_ (%) | 86.2 ± 2.0 | 84.3 ± 2.6 | 85.7 ± 0.9 | 86.0 ± 0.9 | 87.2 ± 1.4 | 86.1 ± 1.8 | 86.6 ± 1.9 | 86.6 ± 1.9 | 86.3 ± 2.4 | 85.8 ± 2.0 | 85.9 ± 2.1 |
| Hb (g/dL) | 9.8 ± 0.7 | 9.3 ± 0.5 | 9.9 ± 0.4 | 10.3 ± 0.3 | 10.1 ± 0.4 | 9.9 ± 0.5 | 9.6 ± 0.6 | 9.7 ± 0.6 | 9.6 ± 0.5 | 9.3 ± 0.4 | 9.4 ± 0.5 |
| Oxygen Content  (mL O_2_/dL) | 11.79 ± 1.0 | 11.1 ± 0.7***** | 12.0 ± 0.5 | 12.5 ± 0.3 | 12.4 ± 0.6 | 12.1 ± 0.8 | 11.7 ± 0.8 | 11.9 ± 0.9 | 11.7 ± 0.7 | 11.3 ± 0.6 | 11.5 ± 0.8 |
| PCO_2_ (mmHg) | 32.8 ± 4.0 | 33.4 ± 3.1 | 32.5 ± 2.7 | 33.0 ± 4.4 | 33.6 ± 3.7 | 34.7 ± 3.4 | 34.3 ± 3.6 | 33.7 ± 3.0 | 33.3 ± 2.8 | 33.1 ± 2.9 | 32.2 ± 2.6 |
| pH | 7.52 ± 0.03 | 7.51 ± 0.02 | 7.52 ± 0.02 | 7.52 ± 0.02 | 7.51 ± 0.02 | 7.51 ± 0.02 | 7.52 ± 0.03 | 7.52 ± 0.03 | 7.52 ± 0.03 | 7.52 ± 0.04 | 7.51 ± 0.03 |
| Lactate (mM) | 0.61 ± 0.20 | 1.09 ± 0.20****** | 1.10 ± 0.29******* | 1.06 ± 0.26****** | 1.07 ± 0.36****** | 1.01 ± 0.34****** | 0.94 ± 0.34 | 0.87 ± 0.42 | 0.77 ± 0.31 | 0.66 ± 0.26 | 0.69 ± 0.28 |

**Supplementary Table 1: Venous blood oximetry and chemistry**

Data are expressed as mean and standard deviation. PO_2_, partial pressure of oxygen, PCO_2_, partial pressure of carbon dioxide, SO_2_ = saturation of haemoglobin with oxygen. Hb, haemoglobin. Blood oxygen content was calculated as (0.0139 X [Hb] X SO_2_) + (0.003 X PO_2_). P_50_ is the partial pressure of oxygen when haemoglobin is 50% saturated with oxygen. It was estimated from PO_2_ and SO_2_, in mixed venous blood, by the method of Doyle.^1^ n = 10 for all mixed venous blood variables. Due to dysfunction of renal venous cannula, n = 7 for all renal venous blood variables. Data were subjected to one-way repeated measures analysis of variance with a Greenhouse-Geisser correction applied to the main effect of ‘time’. Within-animal pairwise comparisons were performed using Dunnett’s test. The baseline measurements were conducted for a period of 30 min. Synthamin® 17 (a proprietary mixture of amino acids) was then infused over the 0 to 30 min period. **P* ≤ 0.05, ***P* < 0.01, ****P* < 0.001 (Dunnett’s test) for comparison with the baseline.


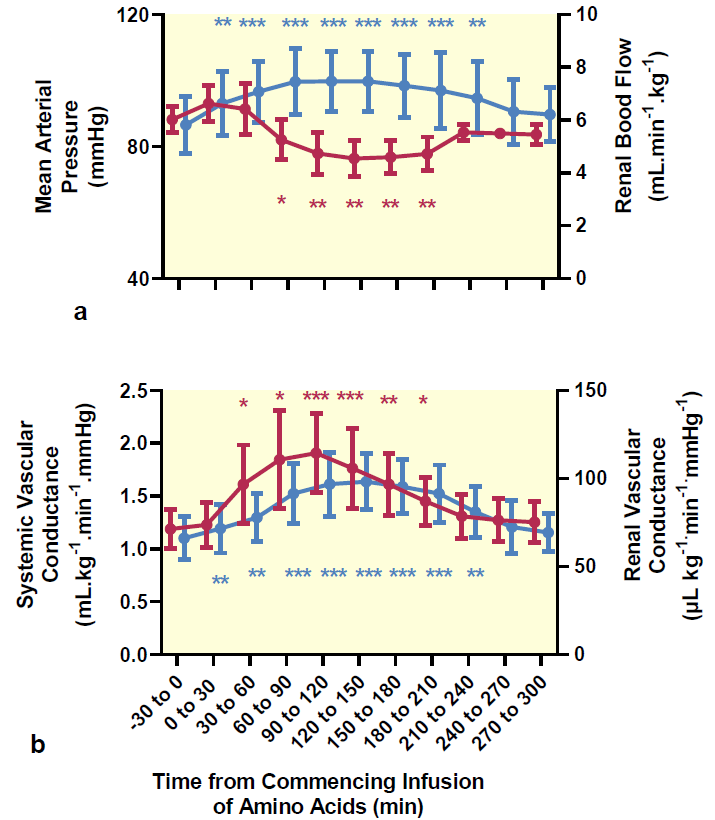


**Supplementary Figure 1.** Time-course of changes in sytemic and renal haemodynamic variables after commencing infusion of a proprietary mixture of amino acids (Synthamin® 17). Error bars represent mean and standard deviation. n = 10 for all except systemic vascular conductance for which n = 7. In Figure 1a, red error bars represent mean arterial pressure and blue bars represent renal blood flow. In Figure 1b, red error bars represent systemic vascular conductance and blue bars represent renal vascular conductance. Data were subjected to one-way repeated measures analysis of variance with a Greenhouse-Geisser correction applied to the main effect of ‘time’. **P* ≤ 0.05, ***P* < 0.01, ****P* < 0.001 (Dunnett’s test) for comparison with the baseline (-30 to 0 min). The amino acids were infused over the 0 to 30 min period.

**Supplementary Figure 2.** Arterial plasma creatinine concentration before, during and after infusion of a proprietary mixture of amino acids (Synthamin® 17). Column and Error bars represent mean and standard deviation (n = 10). The white column represents plasma creatinine concentration at the end of the baseline period (-30 - 0 min). Blue-filled columns represent plasma creatine concentration at the end of each 30 min experimental period after commencing infusion of the amino acid. The amino acids were infused over the 0 to 30 min period. The change in plasma creatinine concentration between successive experimental periods was small (< 5%). Therefore, our measurement of creatinine clearance was unlikely to be confounded by the expansion of the extracellular fluid volume, during and after infusion of the amino acids.

**Reference**

1. Doyle DJ. A simple method to calculate P50 from a single blood sample. *Int J Clin Monit Comput.* 1997;14:109-111.
